# Evaluation of the impact of subepithelial corneal infiltrates on corneal biomechanics after epidemic keratoconjunctivitis

**DOI:** 10.5935/0004-2749.20220061

**Published:** 2025-08-22

**Authors:** Ceyhun Arici, Pinar Sultan, Burak Mergen

**Affiliations:** 1 Department of Ophthalmology, Cerrahpasa School of Medicine, Istanbul University-Cerrahpasa, Istanbul, Turkey; 2 Department of Ophthalmology, Istanbul Research and Training Hospital, Istanbul, Turkey; 3 Department of Ophthalmology, Basaksehir Cam and Sakura City Hospital, University of Health Sciences, Istanbul, Turkey

**Keywords:** Keratoconjunctivitis, Intraocular pressure, Epithelium, corneal, Adrenal cortex hormones, Cyclosporine, Tonometry, ocular, Ceratoconjuntivite, Pressão intraocular, Epitélio corneano, Corticosteroides, Ciclosporina;Tonometria ocular

## Abstract

**Purpose:**

To examine the effect of subepithelial corneal infiltrates on corneal biomechanical properties after epidemic keratoconjunctivitis compared to that in healthy controls.

**Methods:**

The cross-sectional study included consecutive patients with bilateral subepithelial corneal infiltrates after epidemic keratoconjunctivitis and healthy controls. Best corrected visual acuity corneal subepithelial infiltrate scoring Fantes grading scale, and central corneal thickness were measured. Corneal hysteresis corneal resistance factor Goldmann correlated intraocular pressure and corneal compensated intraocular pressure were assessed using an ocular response analyzer.

**Results:**

This study included 66 eyes of 33 patients with subepithelial corneal infiltrates following epidemic keratoconjunctivitis and randomly selected 37 eyes of 37 healthy volunteers. The mean Fantes and CSIS scores were 1.8 ± 0.8 and 2.9 ± 1.3, respectively, in the first involved eyes and 1.3 ± 1.1 and 1.9 ± 1.7, respectively, in the fellow eyes (p=0.009 and p=0.002, respectively). The first (526.1 ± 28.1 µm; p=0.005) and second involved eyes (523.4 ± 38.1 µm; p=0.044) had significantly thinner corneas compared to that in healthy controls (557.0 ± 38.1 µm). While best-corrected visual acuity showed a positive correlation with corneal resistance factor (r=0.363, p=0.045) and corneal hysteresis (r=0.414, p=0.021), corneal subepithelial infiltrate scoring showed a negative correlation with Goldmann correlated intraocular pressure (r=-0.479, p=0.006) and corneal compensated intraocular pressure (r=-0.413, p=0.021).

**Conclusion:**

Eyes with subepithelial corneal infiltrates had significantly thinner corneas compared to that in healthy controls. A positive correlation of the corneal resistance factor and corneal hysteresis with best-corrected visual acuity and a negative correlation of the Goldmann correlated intraocular pressure and corneal compensated intraocular pressure with corneal subepithelial infiltrate scoring should be taken into account when measuring intraocular pressure values in patients with subepithelial corneal infiltrates.

## Abstract (pt)

**Objetivo:**

Examinar o efeito de infiltrados sub-epiteliais corneanos nas propriedades biomecânicas da córnea após ceratoconjuntivite epidêmica, em comparação com controles saudáveis.

**Métodos:**

Este estudo transversal incluiu pacientes consecutivos com infiltrados sub-epiteliais corneanos bilaterais após ceratoconjuntivite epidêmica e controles saudáveis. Foram medidas a melhor acuidade visual corrigida, uma pontuação do infiltrado sub-epitelial da córnea, a escala de graduação de Fantes e a espessura central da córnea. A histerese da córnea, o fator de resistência da córnea, a pressão intraocular correlacionada à tonometria de Goldmann e a pressão intraocular compensada da córnea foram avaliados com o Ocular Response Analyzer.

**Resultados:**

Este estudo incluiu 66 olhos de 33 pacientes com infiltrados corneanos sub-epiteliais após ceratoconjuntivite epidêmica e selecionou aleatoriamente 37 olhos de 37 voluntários saudáveis. As pontuações médias da escala de Fantes e dos infiltrados sub-epiteliais corneanos nos primeiros olhos acometidos foram respectivamente de 1,8 ± 0,8 e 2,9 ± 1,3. Nos olhos contralaterais, foram respectivamente de 1,3 ± 1,1 e 1,9 ± 1,7 (p=0,009 e p=0,002, respectivamente). O primeiro e o segundo olhos envolvidos tinham córneas significativamente mais finas (respectivamente 526,1 ± 28,1 µm; p=0,005 e 523,4 ± 38,1 µm; p=0,044) em comparação com os controles saudáveis (557,0 ± 38,1 µm). Embora a acuidade visual melhor corrigida tenha mostrado uma correlação positiva com o fator de resistência da córnea (r=0,363, p=0,045) e com a histerese da córnea (r=0,414, p=0,021), a pontuação dos infiltrados sub-epiteliais corneanos mostrou uma correlação negativa com a pressão intraocular correlacionada à tonometria de Goldmann (r=-0,479, p=0,006) e com a pressão intraocular compensada da córnea (r=-0,413, p=0,021).

**Conclusão:**

Os olhos com infiltrados corneanos sub-epiteliais tinham córneas significativamente mais finas em comparação com os controles saudáveis. Ao se medirem os valores de pressão intraocular em pacientes com infiltrados sub-epiteliais corneanos, deve-se levar em consideração tanto as correlações positivas do fator de resistência da córnea e da histerese da córnea com a melhor acuidade visual corrigida quanto as correlações negativas da pressão intraocular correlacionada à tonometria de Goldmann e da pressão intraocular compensada da córnea com a pontuação do infiltrado sub-epitelial da córnea.

## INTRODUCTION

Adenoviruses are the most common cause of viral conjunctivitis. Fifty-one distinct human adenoviral serotypes have been described. Epidemic keratoconjunctivitis (EKC) is the most severe ocular disease resulting from adenoviruses^(1)^. Adenoviruses 8, 19, and 37 are the most common serotypes associated with EKC, but many other types have been documented^(2)^.

Subepithelial infiltrates (SEIs) are small, nummular, and grayish lesions. They are lymphocytic infiltrates in the superficial corneal stroma and the overlying epi thelium and represent an immune reaction. These nummuli can impair visual acuity but usually regress in a few weeks and persist for years in rare cases^(3)^. SEIs can lead to corneal scarring that results in permanent loss of vision^(4)^. EKC may be further complicated by lacrimal drainage abnormalities^(5)^, dry eye, and symblepharon formation^(6)^. The involvement of EKC is usually bilateral and asymmetrical. Despite more severe involvement of the first involved eye, tear film functions, and visual acuity were shown to be affected similarly in both eyes^(7)^.

Topical steroids are effective in the treatment of SEIs; however, recurrences may occur after withdrawal, and the patient may become steroid dependent. After long-term treatment with topical steroids, side effects such as increase in intraocular pressure (IOP) and cataracts can develop. Thus, accurate IOP measurement is very important during the treatment of SEIs with topical steroids to prevent optic nerve damage. IOP is the most important and modifiable risk factor in patients with glaucoma. Accurate IOP measurement plays a crucial role in the diagnosis and management of glaucoma^(8)^. Corneal biomechanical properties are among the factors known to influence IOP measurement. Ambrosio et al.^(9)^ reported that corneal biomechanical characteristics have a potential role in the pathogenesis, progression, and diagnosis of ocular diseases, such as keratoconus, iatrogenic ectasia, and glaucoma. Therefore, it is important to evaluate the corneal biomechanics in eyes with SEIs. To our knowledge, to date, no study has investigated the effect of SEIs on corneal biomechanics.

The current study aimed to investigate the effect of SEIs after EKC on corneal biomechanical measurements (corneal hysteresis [CH], corneal resistance factor [CRF], central corneal thickness [CCT]) and compare these mea surements with healthy controls.

## METHODS

Consecutive patients with bilateral SEIs secondary to EKC and healthy controls were included in the study. This cross-sectional study was carried out in accordance with the Helsinki Declaration and was approved by the local ethics committee. Informed consent was obtained from all patients and volunteers included in the study.

The inclusion criterion was having persistent bilateral SEIs in the cornea secondary to EKC. Patients were between 18 and 65 years old, and only those with bilateral involvement were included. Exclusion criteria were as follows: having had a previous ocular surgery, a history of herpes simplex keratitis, corneal scar, dry eye, eyelid disorders, a previous history of contact lens use and glaucoma, cigarette smoking, pregnancy, and breastfeeding.

Patients were divided into the study and control groups. The study group was further divided into two subgroups: the first and the second involved eyes. The control group included the randomly selected eyes of healthy controls.

None of the patients received topical corticosteroids or cyclosporine A (CsA) 1% in the acute stage of conjunctivitis. Loteprednol etabonate and CsA were given to patients with symptomatic SEIs in the chronic phase of the disease. None of the patients was using eye drops. Age, sex, and best corrected visual acuity (BCVA) testing with the logarithm of the minimum angle of resolution (logMAR) measurements were recorded. Detailed slit lamp and fundoscopic examinations were performed. All examinations were performed in the morning to reduce the effects of diurnal variation on the corneal anatomical and biomechanical properties. The corneal subepithelial infiltrate scoring (CSIS) and Fantes grading scale were used to evaluate the severity of corneal involvement. CCT, CH, CRF, corneal compensated intraocular pressure (IOPcc), and Goldmann correlated intraocular pressure (IOPg) were used to evaluate the corneal biomechanics.

CSIS, which varied between 0 and 4, were generated according to the number of SEIs seen in biomicroscopic examinations (0, no infiltrate; 1, 1-5; 2, 6-10; 3, 1-15; 4, >16 infiltrates). Corneal haze due to infiltrates was scored through biomicroscopic examination using the Fantes grading scale: stage 0, clear cornea or no haze; stage 1, trace haze of minimal density seen with difficulty using direct illumination; stage 2, moderate haze easily visible with direct slit illumination; stage 3, marked haze partly obscuring anterior chamber observation or iris details; and stage 4, severe haze obscuring anterior chamber or iris details^(10)^.

CH, CRF, IOPg, and IOPcc were all measured using the ocular response analyzer (software version 2.04, Reichert Ophthalmic Instruments, Buffalo, NY, USA), which works through the noncontact tonometry principle. A rapid puff of air creates an indentation in the cornea and an advanced electro-optical system to record two applanation pressure measurements - one while the cornea moves inward and the other as the cornea moves outward. CH was taken as a measure of viscous properties, whereas the CRF reflects corneal elasticity. IOPg is similar to a standard noncontact tonometry IOP measurement and is affected by the physical properties of the cornea. In contrast, IOPcc is a measurement of pressure based on a mathematical correction and is less affected by corneal properties. To obtain reliable IOP and corneal biomechanical values, measures were taken from the center of the cornea three times and the measure having the highest waveform score was selected for further analysis. CCT was measured with an optical pachymeter (Tonoref III, Nidek, Japan).

### Statistical analysis

Data were statistically analyzed using IBM SPSS Statistics Standard Pack version 21 (licensing type, network; Istanbul University Licensed Software). The chi-square test was used for categorical variables. The sample distribution was evaluated through the Kolmogorov-Smirnov test. The means of the three groups were compared using the analysis of variance and post hoc Tukey test. Those without normal distribution were compared using the Kruskal-Wallis test and post hoc Mann-Whitney U test with Bonferroni correction. The mean CSIS and Fantes scores of the first and second involved eyes (dependent groups) were compared using the Wilcoxon test. The results were presented as mean and standard deviation. Spearman’s correlation coefficient was employed to calculate associations between variables. P<0.05 was considered statistically significant.

## RESULTS

This study included 66 eyes of 33 patients with SEIs following EKC and randomly selected 37 eyes from 37 healthy volunteers. The mean age was 37.0 ± 15.8 (1865) years in patients with SEIs and 38.8 ± 13.9 (18-65) years in the control group (p=0.62). The female to male ratio was 27/6 in the study group and 30/7 in healthy controls (p=0.94).

The mean duration of topical steroid use in the first and second involved eyes were 11.6 ± 14.1 and 10.0 ± 10.3 weeks, respectively (p=0.93). The mean duration of cyclosporine use in the first and second involved eyes was 4.7 ± 8.7 and 4.5 ± 8.3 months, respectively (p=0.98). The mean interval between the development of EKC and examination was 25.5 ± 22.9 weeks.

The mean Fantes score of the first involved eyes was 1.8 ± 0.8 and 1.3 ± 1.1 in the second involved eyes (p=0.009). Similarly, the mean CSIS score was 2.9 ± 1.3 in the first involved eyes and 1.9 ± 1.7 in the second involved eyes (p=0.002).

The mean CCT was 526.1 ± 28.1 µm in the first involved eyes, 523.4 ± 38.1 µm in the second involved eyes, and 557.0 ± 38.1 µm in healthy controls (p=0.003). The post hoc analysis with Bonferroni correction showed that the mean CCT in the first and second involved eyes was significantly lower than that in healthy controls (p=0.005 and p=0.044, respectively) without any significant difference between the first and second eyes (p=1.0). The mean BCVA was 0.20 ± 0.29 in the first involved eyes, 0.16 ± 0.20 in the second involved eyes, and -0.01 ± 0.05 in healthy controls. The post hoc analysis with Bonferroni correction showed that the mean logMAR BCVA in the first and second involved eyes was significantly higher than that in healthy controls (p<0.001) without any significant difference between them (p=1.0).

Despite significant differences in BCVA and CCT, no significant difference was observed among the three groups in terms of the CH, CRF, IOPg, and IOPcc values. The comparison of the means is shown in table 1.

**Table 1:** Comparison of the biomechanical properties of the cornea among the three groups

	First involved eyes	Second involved eyes	Control group	P value
**BCVA**	0.20 ± 0.29	0.16 ± 0.20	-0.01 ± 0.05	<0.001^*^
**CCT**	526.1 ± 28.1	523.4 ± 38.1	557.0 ± 37.9	0.003^*^
**CH**	10.4 ± 1.6	14.0 ± 10.5	11.1 ± 1.6	0.108
**CRF**	10.3 ± 1.9	13.8 ± 10.4	10.9 ± 1.9	0.331
**IOPg**	14.9 ± 4.2	15.3 ± 3.9	15.1 ± 3.6	0.792
**IOPcc**	15.4 ± 4.0	15.8 ± 3.9	14.9 ± 3.4	0.489

BCVA= best corrected visual acuity; CCT= central corneal thickness; CH= corneal hysteresis; CRF= corneal resistance factor; IOPg= Goldman intraocular pressure; IOPcc= CC intraocular pressure.

* p<0.05

The correlation analysis of the biomechanical properties with the SEI scores among the first and second involved eyes is shown in tables 2 and 3, respectively. Among the first involved eyes, BCVA showed a positive correlation with CRF (r=0.363, p=0.045) and CH (r=0.414, p=0.021). CSIS showed a negative correlation with IOPg (r=-0.479, p=0.006) and IOPcc (r=-0.413, p=0.021). CH showed a positive correlation with CCT (r=0.491, p=0.024).

**Table 2:** The correlation of the biomechanical properties with the severity of corneal involvement and the duration of treatment among the first involved eyes in patients with SEIs secondary to epidemic keratoconjunctivitis

		SEI duration	Steroid duration	Cyc duration	BCVA	Fantes	CSIS	IOPcc	IOPg	CRF	CH	CCT
**SEI duration**	**r p**	1										
**Steroid duration**	**r p**	0.781^**^ <.001	1									
**Cyc duration**	**r p**	0.159 0.385	0.314 0.080	1								
**BCVA**	**r p**	-0.253 0.162	-0.226 0.215	-0.220 0.226	1							
**Fantes**	**r p**	-0.252 0.164	-0.026 0.887	0.166 0.365	0.121 0.509	1						
**CSIS**	**r p**	-0.173 0.344	-0.327 0.068	-0.201 0.271	0.190 0.299	0.525^**^ 0.002	1					
**IOPcc**	**r p**	0.160 0.390	0.157 0.399	-0.139 0.455	-0.170 0.359	-0.352 0.052	-0.479^**^ 0.006	1				
**IOPg**	**r p**	0.113 0.545	0.128 0.493	-0.226 0.222	0.028 0.881	-0.268 0.145	-0.413^*^ 0.021	0.905^**^ <0.001	1			
**CRF**	**r p**	-0.016 0.934	0.029 0.879	-0.273 0.138	0.363^*^ 0.045	-0.032 0.862	-0.129 0.488	0.319 0.081	0.690^**^ <0.001	1		
**CH**	**r p**	-0.079 0.674	-0.035 0.852	-0.123 0.511	0.414^*^ 0.021	0.201 0.278	0.140 0.453	-0.363^*^ 0.045	0.034 0.856	0.679^**^ <0.001	1	
**CCT**	**r p**	0.353 0.107	0.391 0.072	0.242 0.277	-0.145 0.519	0.257 0.237	0.100 0.651	-0.273 0.231	-0.056 0.810	0.288 0.206	0.491^*^ 0.024	1

* = p<0.05;

** p<0.01 (two-tailed); r= correlation coefficient.

BCVA= best corrected visual acuity; CCT= central corneal thickness; CH= corneal hysteresis; CRF= corneal resistance factor; IOPg= Goldmann correlated intraocular pressure; IOPcc= corneal compensated intraocular pressure; CSIS= corneal subepithelial infiltrate score; Cyc= cyclosporine; SEI= subepithelial infiltrate

**Table 3:** The correlation of the biomechanical properties with the severity of corneal involvement and the duration of treatment among the second involved eyes in patients with SEIs secondary to epidemic keratoconjunctivitis

		SEI duration	Steroid duration	Cyc duration	BCVA	Fantes	CSIS	IOPcc	IOPg	CRF	CH	CCT
**SEI duration**	**r p**	1										
**Steroid duration**	**r p**	0.724^**^ <0.001	1									
**Cyc duration**	**r p**	0.162 0.376	0.274 0.134	1								
**BCVA**	**r p**	-0.270 0.135	-0.173 0.343	-0.198 0.278	1							
**Fantes**	**r p**	-0.364^*^ 0.041	-0.333 0.063	-0.015 0.937	0.402^*^ 0.022	1						
**CSIS**	**r p**	-0.434^*^ 0.013	-0.465^**^ 0.007	-0.093 0.612	0.411^*^ 0.019	0.879^**^ <0.001	1					
**IOPcc**	**r p**	-0.122 0.513	-0.217 0.241	-0.143 0.444	0.374^*^ 0.038	-0.020 0.914	-0.039 0.833	1				
**IOPg**	**r p**	-0.141 0.448	-0.233 0.207	-0.239 0.195	0.417^*^ 0.020	-0.066 0.725	-0.050 0.788	0.926^**^ <0.001	1			
**CRF**	**r p**	-0.152 0.415	-0.183 0.324	-0.303 0.098	0.313 0.086	-0.106 0.521	0.020 0.917	0.312 0.087	0.639^**^ <0.001	1		
**CH**	**r p**	-0.051 0.786	-0.008 0.966	-0.157 0.398	-0.025 0.896	-0.090 0.630	0.064 0.733	-0.462^**^ 0.009	-0.107 0.566	0.686^**^ <0.001	1	
**CCT**	**r p**	0.449^*^ 0.032	0.464^*^ 0.026	0.251 0.248	0.226 0.300	-0.132 0.537	-0.168 0.432	-0.066 0.771	0.011 0.962	0.102 0.653	0.134 0.551	1

* = p<0.05;

** = p<0.01.

BCVA= best corrected visual acuity; CCT= central corneal thickness; CH= corneal hysteresis; CRF= corneal resistance factor; IOPg= Goldmann correlated intraocular pressure; IOPcc= corneal compensated intraocular pressure; CSIS= corneal subepithelial infiltrate score; Cyc= cyclosporine; SEI= subepithelial infiltrate.

The correlation analysis among the second involved eyes resulted in a positive correlation between CCT and the duration of SEI (r=0.449, p=0.032) or the duration of steroid use (r=0.464, p=0.026). Regression analysis showed that CCT has no significant linear relationship with the duration of SEI (t=0.76, p=0.456) or the duration of steroid use (t=0.96, p=0.349). BCVA showed a positive correlation with IOPcc (r=0.374, p=0.038) and IOPg (r=0.417, p=0.02).

## DISCUSSION

Conjunctival infection due to adenovirus is the most common external ocular infection worldwide^(11)^. After an incubation period of 2 to 14 days, ocular symptoms can appear in one eye followed by the involvement of the second eye a few days later^(3)^. SEIs can develop and persist for months or even years in patients with EKC. Although usually minimally symptomatic, these infiltrates can cause significant ocular morbidity, reduced vision, photophobia, glare, halos, and foreign body sensation^(12)^. Since topical steroids are used for the treatment of SEIs, careful monitoring of the IOP is crucial. To obtain an accurate measurement, the corneal biomechanical change should be investigated in eyes with SEIs. Thus, in this study, we examined the difference in the corneal biomechanics and found that eyes with SEIs had significantly lower CCT compared to that in healthy controls. Additionally, among the first involved eyes, BCVA showed a positive correlation with CRF and CH, whereas CSIS showed a negative correlation with IOPg and IOPcc suggesting a significant effect of SEIs on the corneal biomechanics.

SEIs usually resolve with topical steroid treatment but recur when steroids are discontinued^(13)^. We used topical cyclosporine as a steroid-sparing agent for mild recurrences of symptoms caused by SEIs^(14)^, to maintain corticosteroid-induced remissions in selected cases. However, while using steroids, accurate measuring of the IOP is very important. Accurate measurement of the IOP depends on the corneal biomechanical properties including the central corneal thickness. There is a good consensus between studies showing the correlation of a decreased IOP measurement with decreased CCT^(15-17)^. Thus, eyes with thinner cornea need correction toward a higher IOP level. In our study, both the first and second eyes with SEIs had significantly lower CCT compared to that in healthy controls. Therefore, we concluded that CCT should be taken into account during IOP measurement. However, the mechanism of corneal thinning is unclear. In vivo confocal microscopy studies revealed that immune response to corneal intraepithelial adenoviral infection includes the recruitment of dendritic cells initially as innate immunity followed by an anterior stromal reaction at the second week compatible with SEIs^(18,19)^. The stromal reaction was thought to include keratocytes, highly active myofibroblasts, and infiltrated inflammatory cells^(18,20)^. Besides, midstromal infiltrates in form of hyperreflective plaques compatible with the healing process were observed in as late as 24 weeks. This wound healing process with possible transdifferentiation of keratinocytes into myofibroblasts might be responsible for the corneal thinning observed in our patient cohort. Among the second involved eyes, CCT was positively correlated with the duration of SEI, suggesting that CCT might decrease when SEIs first appeared followed by an increase in time.

In our study, despite no difference in the CRF and CH between the eyes with SEIs and healthy controls, BCVA showed a positive correlation with CRF and CH among the first involved eyes. Since BCVA was represented as logMAR, decreased visual acuity was correlated with increased CRF and CH. The positive correlation of CRF and CH with BCVA rather than Fantes and CSIS scores can be explained with the significance of the SEI density than that of the number of SEIs. The eyes with denser SEIs causing lower BCVA might have affected the corneal biomechanics. However, prospective studies following patients with SEIs at multiple visits are needed for a better understanding of the effect of SEIs on corneal biomechanics in time. While CH reflects viscous properties, the CRF reflects corneal elasticity. Therefore, a lower BCVA was correlated with increased corneal viscosity and elasticity that might lead to overestimation of the IOPg measurement. Conversely, CSIS showed a negative correlation with both IOPcc and IOPg, suggesting that having a higher number of infiltrates might lead to underestimation of the IOP measurement. Because of this conflicting effect of SEIs on corneal biomechanics, a subgroup analysis for the eyes with denser SEIs or with high CSIS scores should be compared with the healthy control in further studies. The finding of no difference in the CRF and CH might also be explained by the heterogeneous patient group.

Additionally, CCT was positively correlated with the duration of topical cyclosporine use and the duration of SEIs in the second involved eyes. Topical cyclosporine or tacrolimus treatment (calcineurin inhibitors) usually ameliorates the corneal opacities that result from an immunologic host reaction^(21)^. This response to topical steroids in patients with SEIs may be attributed to its effect on the corneal stroma as well as bias due to the longer follow-up in these patients. A similar correlation was not observed in the first involved eye of patients with EKC with SEIs. The reason for this may be a more severe clinical course and more resistance of SEIs to treat ment in the first involved eye of EKC compared to that in the second involved eye. In our study, first involved eyes showed higher Fantes and CSIS scores compared to that in the fellow eyes. Further studies are needed to investigate the pathophysiology of the changes in corneal tissue secondary to SEIs.

In EKC, SEIs may progress in up to half of patients and about half of those with unilateral involvement^(22)^. In our study, the second eyes in all patients were affected 2 to 7 days after the disease appeared in the first eye. The Fantes grading and CSIS scales revealed that the mean number of corneal infiltrates and corneal haze were significantly higher in the first involved eyes compared to that in the second eye. However, BCVA showed no difference between the two eyes despite reduced BCVA compared to healthy controls. In addition, no difference in corneal biomechanical properties was observed. Although a higher number of corneal infiltrates and corneal haze were observed in the first involved eyes, these differences were not sufficient to change corneal biomechanics. In contrast, compared to the control group, BCVA was affected in the first involved eye. Appro ximately 10 days after the onset of symptoms of EKC, SEIs can develop and persist for months or even years^(23)^. In the current study, the mean interval between EKC and the examination was 25.5 ± 22.9 weeks.

The limitations of the study include its cross-sectional design and lack of subgroup analysis of the eyes with different CSISs. Further studies involving a higher number of patients should be conducted to analyze the effect of SEI density in detail.

In conclusion, the eyes with SEIs after EKC had significantly lower CCT compared to that in healthy controls. While BCVA showed a positive correlation with CH and CRF, CSIS showed a negative correlation with IOPg and IOPcc. Additionally, although the first involved eyes of EKC with SEIs was denser than the second eye, the corneal biomechanical properties were observed to be similar. The effect of SEIs on corneal biomechanics was complex, but the changes should be kept in mind while measuring the IOP, especially during topical steroid treatment. However, further prospective studies are needed to determine the real impact of SEIs.
